# Targeting the central and peripheral nervous system to regulate bone homeostasis: mechanisms and potential therapies

**DOI:** 10.1186/s40779-025-00600-8

**Published:** 2025-03-20

**Authors:** Tong-Zhou Liang, Zhe-Yu Jin, Yue-Jun Lin, Zi-Yi Chen, Ye Li, Jian-Kun Xu, Fan Yang, Ling Qin

**Affiliations:** 1 Musculoskeletal Research Laboratory of Department of Orthopaedics & Traumatology and Innovative Orthopaedic Biomaterial and Drug Translational Research Laboratory, Li Ka Shing Institute of Health, the Chinese University of Hong Kong, Sha Tin, 999077 Hong Kong China; 2 Innovative Orthopedic Biomaterial and Drug Translational Research Laboratory, Li Ka Shing Institute of Health Sciences, Prince of Wales Hospital, the Chinese University of Hong Kong, Sha Tin, 999077 Hong Kong China; 3 The Brain Cognition and Brain Disease Institute (BCBDI), Shenzhen Institute of Advanced Technology, Chinese Academy of Sciences, Shenzhen-Hong Kong Institute of Brain Science-Shenzhen Fundamental Research Institutions, Shenzhen, 518055 Guangdong China; 4 Areas of Excellence Centre for Musculoskeletal Degeneration and Regeneration, Sha Tin, 999077 Hong Kong China

**Keywords:** Osteoporosis, Central nervous system, Brain nuclei, Peripheral nerve fiber, Bone homeostasis

## Abstract

The skeleton is innervated by different types of nerves and receives signaling from the nervous system to maintain homeostasis and facilitate regeneration or repair. Although the role of peripheral nerves and signals in regulating bone homeostasis has been extensively investigated, the intimate relationship between the central nervous system and bone remains less understood, yet it has emerged as a hot topic in the bone field. In this review, we discussed clinical observations and animal studies that elucidate the connection between the nervous system and bone metabolism, either intact or after injury. First, we explored mechanistic studies linking specific brain nuclei with bone homeostasis, including the ventromedial hypothalamus, arcuate nucleus, paraventricular hypothalamic nucleus, amygdala, and locus coeruleus. We then focused on the characteristics of bone innervation and nerve subtypes, such as sensory, sympathetic, and parasympathetic nerves. Moreover, we summarized the molecular features and regulatory functions of these nerves. Finally, we included available translational approaches that utilize nerve function to improve bone homeostasis and promote bone regeneration. Therefore, considering the nervous system within the context of neuromusculoskeletal interactions can deepen our understanding of skeletal homeostasis and repair process, ultimately benefiting future clinical translation.

## Background

Bone is a dynamic organ that constantly undergoes new bone formation and bone resorption. In the process of maintaining bone homeostasis, three major cell types within bone are regarded as most important. Osteoblasts mediate bone formation and osteoclast drive bone resorption, while osteocytes can control the activity of both osteoclast and osteoblast and maintain bone structural integrity [1]. Bone homeostasis is tightly controlled by internal and external clues, including hormones, mechanical loading and systemic factors [2, 3]. Disruption of this equilibrium underlies pathologies like osteoporosis and fracture healing delay, highlighting the clinical significance of understanding bone metabolism process [4, 5].

Osteoporosis (OP) is one of the most prevalent orthopedic diseases in the world, and it represents the most classic abnormal bone metabolism disease. According to the World Health Organization, it is estimated that the global prevalence of OP is about 18.3% [6]. This disease is characterized by bone fragility and an increased susceptibility to fractures, particularly in the hip, spine, and wrist. Primary OP, which is mainly associated with aging, affects bone quality. Postmenopausal OP, the most common primary OP, results from decreased estrogen levels in women. Although postmenopausal OP is the most prevalent type of primary OP, men are also susceptible to age-related OP, which is mainly characterized by reduced cortical thickness [7]. Secondary OP is caused by various medical conditions and medications. For example, epilepsy and antiseizure medications were recognized as independent risk factors for secondary OP [8]. In addition to OP, other musculoskeletal disorders including osteoarthritis (OA) and rheumatoid arthritis (RA) might develop bone metabolism disorders, such as subchondral bone stiffness in OA [9] and bone destruction in RA. Both OA and RA patients develop chronic pain in the late stage, and pain is often the major complaint and may lead to exacerbation of the disease [10, 11].

The nervous system plays a central role in sensing and regulating the homeostasis of the body. The intricate interplay between the nervous system and peripheral organ functions has been identified. Manipulating the brain and efferent nerves exerted direct and reciprocal effects on various peripheral organs, including the liver [12], heart [13], intestine [14], adipose tissue [15], immune cells [16], and respiratory tract [17]. The skeletal system, which functions as both a structural support for the body and a niche for hematopoiesis, is densely innervated by nerves in the periosteum, bone marrow, and growth plate [18]. Impairment or enhancement of nerve function is closely associated with bone mineral density (BMD) and fracture healing. Given the important role of the nervous system in bone metabolism, particularly in OP and bone fracture, novel mechanisms and strategies targeting the nervous system may offer promising avenues for the treatment of bone disease [19, 20].

## Functional central nervous system (CNS) connections

**Fig. 1:**
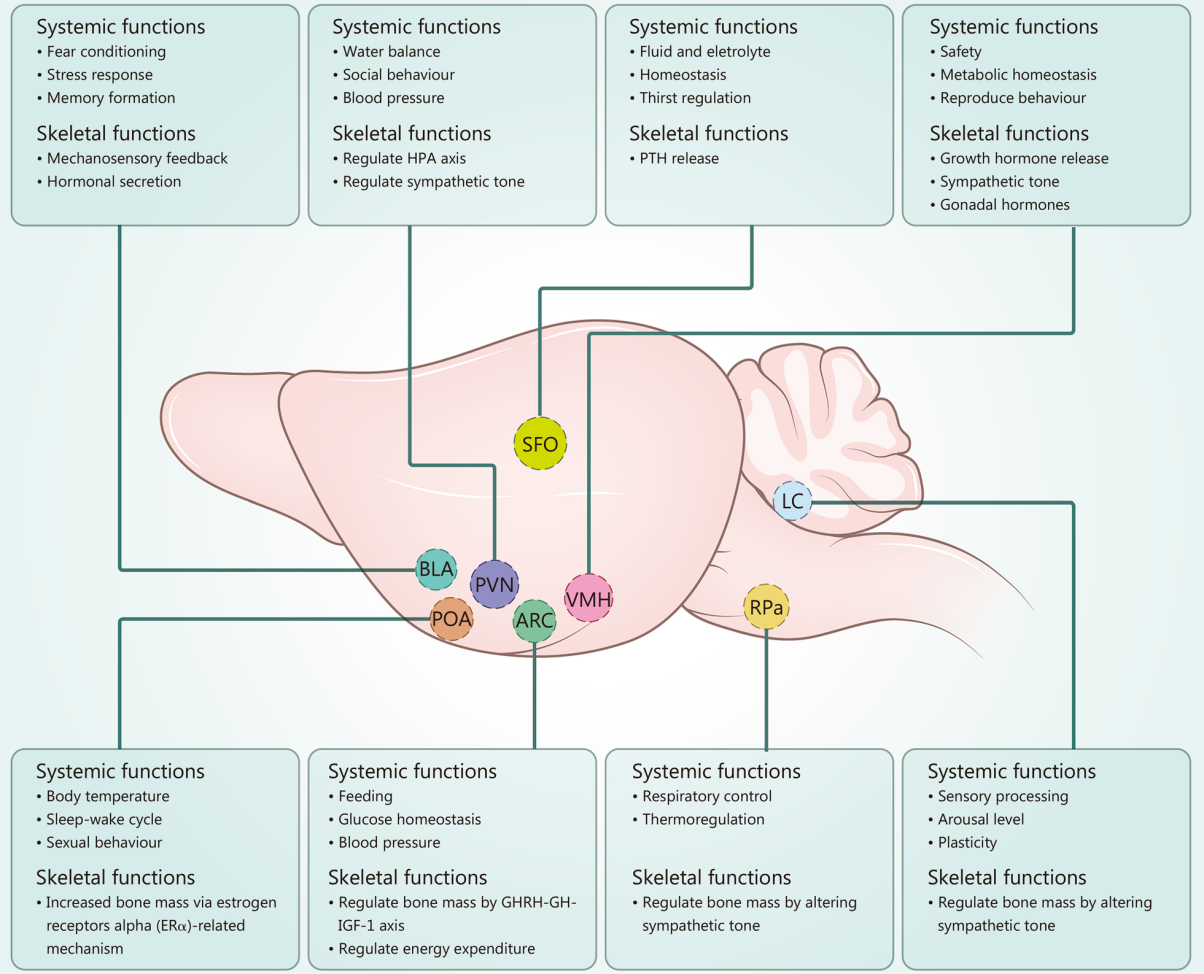
Control of bone metabolism by central nervous system regions. Brain nuclei can control different aspects of the body and bone homeostasis. These nuclei are associated with some very fundamental functions of the body, including fluid and electrolyte control, circulation function, general arousal level, etc. These nuclei can also regulate bone mass by altering the bone-regulating hormone (PTH, gonadal hormone, growth factor, and epinephrine) level or response to the hormone. These brain nuclei may also send nerve projections to bone and regulate bone homeostasis via the release of neurotransmitters or neuropeptides. SFO subfornical organ, LC locus coeruleus, BLA basolateral complex of the amygdala, PVN paraventricular hypothalamic nucleus, POA preoptic area, ARC arcuate nucleus, VMH ventromedial hypothalamus, RPa raphe pallidus nucleus, HPA hypothalamic–pituitary–adrenal, PTH parathyroid hormone, GHRH growth hormone-releasing hormone, GH growth hormone, IGF-1 insulin-like growth factor-1

Brain nuclei are responsible for distinct biological processes. Historically, the identification of regions governing specific biological functions has relied on observations made by neurosurgeons. With advancements in neuroscience, it is now possible to precisely locate brain nuclei that are essential in organ homeostasis (Fig. 1). This review will focus on the evidence that links a specific brain nucleus with bone metabolism and will discuss the underlying mechanism (Table 1) [21–39].

**Table 1:** Bone density regulated by central nervous system regions and neuron populations

Brain region	Neuron subtype	Manipulation method	Effects on bone	References
ARC	AgRP	*Sirt1*, *Ucp2* gene deletion; Neurons-specific deletion by diphtheria toxin	Reduced trabecular bone mass	[21]
		*AgRP* gene deletion	Increased cortical and trabecular bone mass	[22]
		*AP1* gene alterations	Increased cortical and trabecular bone mass	[23]
		*PDK1* gene deletion	Reduced cortical and trabecular bone mass	[24]
	Kiss	*ERα* gene deletion	Increased trabecular bone mass	[25]
	POMC	*ERα* gene deletion	Increased cortical bone mass	[26]
		AP1 alterations	Increased cortical and trabecular bone mass	[25]
	NPY	NPY overexpression	Decreased bone mass	[27]
	NTS	*NPY* knockout	Reduced cortical bone mass, but unchanged trabecular bone mass	[28]
	NPY	AP1 alterations	Increased trabecular bone mass	[29]
		*RANK* gene deletion	Rescued bone loss induced by OVX	[30]
	CART	AP1 alterations	Increased trabecular bone mass	[29]
VMH	SF1	CaMK signaling defect; *Creb* gene deletion	Decreased trabecular bone mass	[31]
		*Htr2c* gene deletion	Decreased trabecular bone mass	[32]
	GABAergic neural circuitry	Activation of GABAergic projections	Increased trabecular bone mass	[33]
	SF1	*IFT88* gene deletion	Increased trabecular bone mass	[34]
		*AP1* gene alterations	Decreased cortical and trabecular bone mass	[23]
Hypothalamus and preoptic area	Nkx2-1	*ERα* gene deletion	Increased cortical and trabecular bone mass	[35]
LC, Rpa, PVH	PDE5A	Pharmaceutical activation by tadalafil and vardenafil	Increased trabecular bone mass	[36]
LC	Whole population	*FoxO1* and adiponectin gene deletion	Reverse bone mass loss in FoxO1 and adiponectin^−/−^ mice	[37]
		*Sirt1* deletion	Increased trabecular bone mass	[38]
BLA	CaMKII	Chemogenetic activation	Increased trabecular bone mass	[39]

*ARC* arcuate nucleus, *AgRP* agouti-related peptide, *AP1* activator protein 1, *PDK1* pyruvate dehydrogenase kinase 1, *ERα* estrogen receptor alpha, *POMC* proopiomelanocortin, *NPY* neuropeptide Y, *NTS* nucleus of the solitary tract, OVX ovariectomy, *CART* cocaine- and amphetamine-regulated transcript, *SF1* steroidogenic factor 1, *VMH* ventromedial hypothalamus, *GABA* gamma-aminobutyric acid, *IFT88* intraflagellar transport 88, *LC* locus coeruleus, *Rpa* raphe pallidus, *Nkx2-1* NK2 homeobox 1, *PDE5A* phosphodiesterase 5A, *PVH* paraventricular nucleus of the hypothalamus, *BLA* basolateral complex of the amygdala, *CaMKII* calcium/calmodulin-dependent protein kinase II, *FoxO1* forkhead box O1, *Sirt1* sirtuin 1, *Ucp2* uncoupling protein 2*, RANK* receptor activator for nuclear factor-κ*, Creb* cAMP-response element binding protein*, Htr2c* 5-hydroxytryptamine receptor 2C

### Ventromedial hypothalamus (VMH)

VMH is a distinct hypothalamus nucleus that regulates multiple biological processes, such as energy balance [40], emotion [41], and activity status [42]. Steroidogenic factor-1 (SF1) positive neurons are highly concentrated in the VMH and are thus considered specific markers of this region [43]. These neurons responded to leptin stimuli, thereby maintaining normal body weight homeostasis [44]. Conditional deletion of leptin receptors (LepRs) in SF1-Cre mice resulted in a phenotype resistant to diet-induced obesity.

Increasing evidence showed that VMH is critical in bone metabolism. VMH mediated unloading-induced bone loss. Compared to intact mice, destroying the VMH region via gold-thioglucose injection not only decreased bone mass but also abolished the effect of unloading-induced bone loss [45]. Intriguingly, a study by Yadav et al. [32] found that brainstem-derived serotonin increased bone mass via binding to the 5-hydroxytryptamine receptor 2C (Htr2c) receptor, while leptin serves as an antagonist by reducing serotonin synthesis. The same team further identified cyclic adenosine monophosphate (cAMP) response element-binding protein (CREB) as a signaling pathway regulating bone mass in VMH neurons [31]. Another study revealed that alternation in transcriptional factor activator protein 1 (AP1) in the VMH region reduced bone mass independently of elevated energy metabolism [23]. Hypothalamic primary cilia are functional organelles involved in body homeostasis. Notably, loss of the key cilia gene intraflagellar transport 88 (IFT88) in the VMH region resulted in increased bone mass [34].

In addition to systemic hormone signaling pathways, the VMH also responded to emotional status, thereby affecting bone mass. Chronic stress is a significant factor contributing to decreased bone density [46]. A mouse model of chronic stress replicated the bone loss phenotype observed in crew members. A GABAergic neural circuit projecting from the bed nucleus of the stria terminalis to the VMH mediated this effect. Optogenetic activation of this GABAergic circuit could induce bone loss even in the absence of stress [33]. Furthermore, astrocytes in the VMH region could regulate anxiety-related behavior and bone mass by interacting with SF1 neurons in the VMH [47].

### Arcuate nucleus (ARC)

ARC is a functional-related region of the hypothalamus located above the VMH region. Two major populations of neurons have been extensively studied in the ARC: agouti-related peptide (AgRP) neurons and proopiomelanocortin (POMC) neurons. Functionally, the AgRP neurons promote hunger (or orexigenic), while POMC neurons inhibit hunger (or anorexigenic).

AgRP-positive neurons are exclusively localized in the ARC. Using multiple genetic modification methods, AgRP neurons have been found to regulate bone mass. Deletion of the *Ucp2* and *Sirt1* gene impaired AgRP circuitry, resulting in reduced bone mass. Suppression of sympathetic tone by propranolol can successfully rescue the osteopenia phenotype in AgRP-impaired mice [23]. Leptin and insulin acted on AgRP neurons via an important signaling molecular pyruvate dehydrogenase kinase 1 (PDK1). AgRP-specific deletion of *PDK1* not only decreased bone mass but also impaired bone strength and shortened femur length [24]. Other external factors also participated in regulating AgRP neurons, such as neuropeptide Y (NPY), a neuropeptide that co-localized with AgRP [48]. Similar to the role of orexigenic AgRP neurons, NPY neurons could increase bone mass upon activation by targeted AP1 antagonists [29]. However, a recent study found that *AgRP* gene deletion in both male and female mice exhibited increased cortical and trabecular bone density [22], suggesting a possible bi-directional regulatory role of AgRP neurons.

POMC neurons were predominantly located in the ARC [49]. Idelevich et al. [23] conducted in-depth research into the roles of both POMC and AgRP neurons in regulating bone density. Their research demonstrated that antagonism of AP1 in both POMC and AgRP neurons led to elevated bone density, specifically improving both cortical and trabecular bone. Additionally, estrogen has been shown to act on the ARC region to modulate bone mass. Specifically, the selective knockout of estrogen receptors in POMC neurons resulted in enhanced bone mass in ovariectomy (OVX) induced osteoporotic mice [26].

### Paraventricular hypothalamic nucleus (PVN)

PVN is a distinct region that is located anterior to the ARC. Oxytocin and arginine vasopressin (AVP) are two peptides synthesized by neurons in the PVN and supraoptic nucleus, which are subsequently transported to the posterior pituitary for release [50]. Notably, oxytocin and AVP neurons also project to the forebrain and spinal cord [51], indicating that in addition to their endocrine functions, these peptides played roles in specific neuronal circuits. Various brain regions received oxytocinergic projections, including ARC, VMH, and parabrachial nucleus [52, 53]. Both oxytocin and AVP have been shown to regulate skeletal homeostasis and sympathetic activity. Oxytocin mainly functioned as an anabolic bone regulator [54], while AVP exerted catabolic effects on bone [55]. Oxytocin mediated its effect on bone through its receptor—oxytocin receptor, although it also exhibited affinity for the AVP receptor. Locally, systemic oxytocin treatment improved bone quality by decreasing bone turnover [56]. Global oxytocin knockout or osteoblast-specific oxytocin receptor knockout in both female and male mice resulted in decreased bone mass [54]. Moreover, the PVN received GABAergic projections from the subfornical organ, a recently identified brain nucleus responsible for parathyroid hormone sensation [57]. Chemogenetic activation of excitatory glutamatergic neurons upregulated bone mass via the sympathetic nervous system (SNS) [57].

### Amygdala

Amygdala, a brain region situated in the temporal lobe, includes two major nuclei: the basolateral complex of the amygdala (BLA) and the central nucleus of the amygdala (CeA) [58]. The amygdala has long been recognized as a key structure involved in various emotional responses, such as fear, anxiety, and aggression. Clinical observation utilizing ^18^F-FDG PET/CT has demonstrated that increased metabolic activity in the amygdala was associated with reduced BMD in postmenopausal OP [59]. Deletion of the NPY receptor 2 (Y2R) in the amygdala could increase bone mass in mice, and selective Y2R antagonist treatment led to improvements in bone quality [60]. A direct anatomical connection has been observed between the musculoskeletal system and the amygdala: the femur is linked to the BLA, while the tibialis anterior is associated with the CeA. Chemogenetic activation of calcium/calmodulin-dependent protein kinase II neurons in the BLA region positively regulated bone mass. More importantly, the functional integration between CeA and BLA suggested that muscle mechanosensory feedback regulated bone mass via a CNS-dependent mechanism [39].

### Locus coeruleus (LC)

LC is an important brain nucleus located deep within the brainstem and serves as a major noradrenergic hub for the CNS [61]. LC participates in forming the ascending reticular activating system, which is closely associated with sleep–wake cycles. Several interesting studies have established a link between LC and bone metabolism. For example, a study by Kajimura et al. [37] demonstrated that adiponectin decreases bone mass when it acts on osteoblasts. However, such effects were abolished over time because of the central action of adiponectin on the LC, which reduced sympathetic tone. Global deletion of adiponectin and specific deletion of forkhead box O1 (FoxO1) in the LC reversed the low bone mass phenotype observed in adiponectin global knockout mice, suggesting that FoxO1 was a part of the adiponectin signaling pathway in the LC [37]. Moreover, sympathetic neurons in the LC projected directly to the skeletal system and expressed phosphodiesterase 5A, the target enzyme for erectile dysfunction medications [62]. These anatomical and molecular bases enabled a balance between the central and peripheral effects of phosphodiesterase 5A inhibitors on bone metabolism [62]. Furthermore, sirtuin 1 (Sirt1), a transcriptional modulator, has been found to regulate catecholamine synthesis in the LC by modulating serotonin levels, thereby influencing bone mass [38].

## Peripheral innervation in the skeletal system

### Sensory nerves

The sensory nerve terminals of the axial and appendicular skeleton originate from the dorsal root ganglion (DRG) along the spinal cord, while those of the cranial skeleton arise from the cranial nerve ganglia. Sensory neurons and their fibers are categorized based on size, myelination status, and conduction velocity [63]. Recent advancements in single-cell RNA sequencing contributed to the mechanism underlying the division of sensory nerves from the DRG. Generally, DRG sensory neurons could be classified into the following characteristic subtypes: C-low threshold mechanoreceptors, Aβ-nociceptor, Aδ-nociceptors, cold nociceptors, and proprioceptors [64, 65]. Peptide‐rich fibers expressing markers such as calcitonin gene-related peptide (CGRP) and substance P (SP) are widely distributed throughout the bone. In contrast, peptide-poor C fibers, which express markers like MAS-related G-protein coupled receptor D (Mrgprd), purinergic receptor P2X 3 (P2X3), or isolectin B4 (IB4), showed very limited expression in either mineralized bone or the periosteum [66, 67].

CGRP is a neuropeptide belonging to the calcitonin family and exerts its effect through a heteromeric receptor complex consisting of a calcitonin receptor-like receptor and a receptor activity-modifying protein. Staining for nerves in bone tissue has identified CGRP expression in mineralized bone, bone marrow, and periosteum, confirming the presence of Aβ, Aδ, and C fibers in bone [67, 68]. The majority of CGRP-positive fibers were unmyelinated or thinly myelinated sensory nerve fibers [69].

SP, a member of the tachykinin family, mainly binds to the neurokinin 1 receptor (NK1R). SP is widely expressed in the sensory nerves innervating bone tissue [70]. Similar to CGRP, SP exhibited a comparable distribution pattern near the epiphyseal plate, within the bone marrow, and in the periosteum [68, 71]. The majority of small, unmyelinated SP-positive nerve fibers were predominantly observed around the blood vessels.

Tropomyosin receptor kinase A (TrkA) is another molecule expressed in sensory fibers, and it is also presented in tyrosine hydroxylase (TH)-positive sympathetic fibers [69]. TrkA, together with p75, served as the receptor for nerve growth factor (NGF). Immunohistochemical staining revealed TrkA-positive nerve fibers are observed in the periosteum, bone marrow, and NGF-positive blood vessels, but not in subchondral bone or cartilage [69, 72]. TrkA was extensively expressed in both myelinated and unmyelinated sensory nerve fibers marked by co-expression of NF200 and CGRP, respectively [69].

### Autonomic nervous system

The autonomic nervous system is the functional connection between the CNS and peripheral organs, regulating the internal state of the whole body and responding to both internal and external signals. Two major systems, namely the SNS and the parasympathetic nervous system (PSNS), which together form a mutually antagonistic regulatory network.

#### Sympathetic nerve

**Fig. 2:**
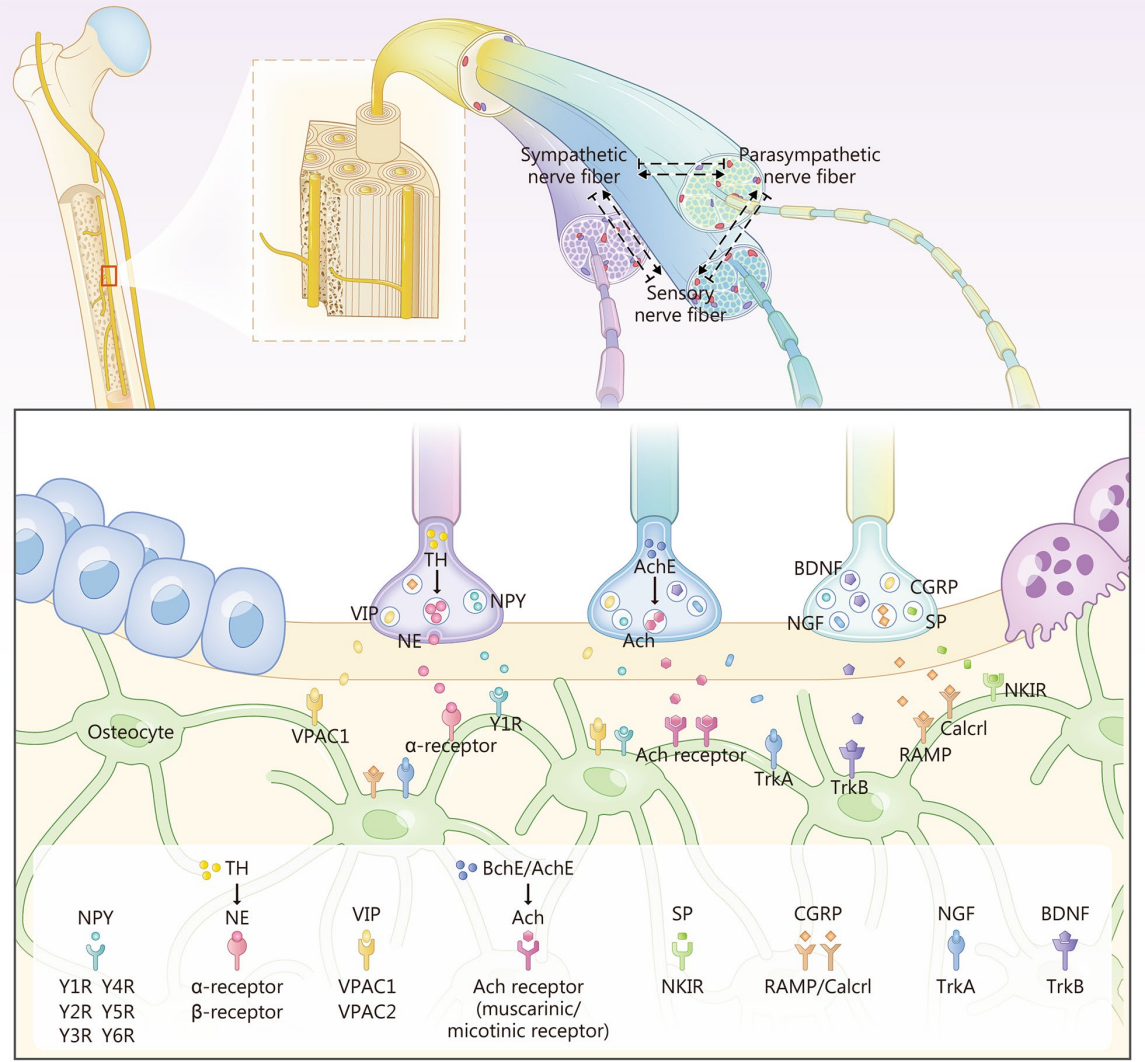
Key biomarkers and regulatory factors released from the nerve terminal in the bone. For sympathetic nerve terminals, tyrosine hydroxylase (TH) is the rate-limiting enzyme for norepinephrine (NE) synthesis and is commonly used as a marker for the sympathetic nerve. For parasympathetic nerve terminals, acetylcholinesterase (AChE) is essential in producing acetylcholine (Ach). Several neuropeptides (BDNF, CGRP, NGF, and SP) can be released from sensory nerve terminals and act on their receptor on target organs. VIP vasoactive intestinal peptide, NPY neuropeptide Y, BDNF brain-derived neurotrophic factor, NGF nerve growth factor, CGRP calcitonin gene-related peptide, SP substance P, VPAC vasoactive intestinal peptide receptor, BchE butyrylcholinesterase, Y1R neuropeptide Y1 receptor, TrkA tropomyosin receptor kinase A, TrkB tropomyosin receptor kinase B, RAMP receptor activity modifying protein, NKIR neurokinin 1 receptor, Calcrl calcitonin receptor like receptor

Preganglionic sympathetic neurons are located in the spinal cord and form synaptic connections with the postganglionic neurons situated in the sympathetic chain. Norepinephrine (NE) is the major neurotransmitter released by postganglionic sympathetic fibers. Dopamine β-hydroxylase and TH are key enzymes involved in NE synthesis. The expression of TH-positive nerve fibers is commonly used to visualize sympathetic nerve fibers. TH-positive nerve fibers were extensively distributed in bone, bone marrow, and periosteum of long bone and vertebral bodies [19, 73, 74]. These fibers were particularly abundant in the vicinity of the epiphyseal plate and Volkmann canals [75]. Similar to sensory nerve fibers, TH-positive sympathetic nerves were frequently colocalized with blood vessels, thus suggesting their role in regulating vascular function in bone [76] (Fig. 2).

NPY is a neuropeptide produced by sympathetic neurons within the peripheral nervous system. NPY-positive nerve fibers were widely distributed in the periosteum, bone marrow, and epiphyseal plate. These nerve fibers were largely confined to the vascular system and among the bone lineage cells [71, 75]. However, some non-vascular NPY-positive fibers were also observed near the growth plate and within the bone marrow [77].

The majority of sympathetic nerves consisted of noradrenergic nerve fibers. However, in addition to the classic noradrenergic sympathetic nerve terminals, a small proportion of cholinergic nerves were also present within the sympathetic system [78]. These sympathetic cholinergic nerves originally possessed noradrenergic activity and then switched to cholinergic postnatally [19]. In the sternum, sympathetic axons exhibited noradrenergic properties during elongation, but the immunoactivity disappeared once the axon reached their target [79]. Alternatively, these axons became cholinergic, marked by vasoactive intestinal peptide (VIP) and vesicular ACh transporter (VAChT) expression on E18. A recent study investigated the molecular mechanisms underlying this neurotransmitter switch. The ablation of the sympathetic nerve resulted in a decrease of cholinergic fibers [GDNF family receptor alpha (GFRa2)-positive or VAChT-positive], indicating that these cholinergic fibers in bone had a sympathetic origin [80].

#### Parasympathetic nerve

Acetylcholine (ACh) acts as the neurotransmitter for parasympathetic neurons. ACh is synthesized by choline acetyltransferase and is loaded into the synaptic vesicles via the VAChT [81]. Parasympathetic nerves originated from specific cranial nerves, the vagus nerve, and the pelvic splanchnic nerves [82]. Staining for markers of parasympathetic nerve fibers has revealed the presence of VAChT-positive [83, 84], and choline acetyltransferase-positive [85] nerve fibers in the periosteum, bone marrow, and metaphysis. However, compared to sympathetic nerves, the parasympathetic nerve is relatively short and sparse. More importantly, the precise pattern and origin of parasympathetic cholinergic innervation remained largely unclear.

## Efferent signaling and their regulatory role in bone remodeling and fracture healing

### Sensory nerve

#### CGRP

CGRP is encoded by the *Calca* gene, which also encodes calcitonin through alternative splicing. Deletion of the *Calca* gene led to an unexpectedly high bone volume phenotype [86]. Compared with *Calca* deletion, CGRP-deleted mice exhibited an osteopenia phenotype, which is possibly the result of a reduced bone formation rate [87]. In vitro experiments demonstrated that the CGRP promoted osteogenesis in bone marrow stromal cells or osteoblasts [88, 89]. The expression level of CGRP elevated in the animal model of OVX-induced OP [90]. In osteoporotic mice, osteoclasts promoted the release of CGRP. However, the secretion was counteracted by the activation of the descending serotonergic inhibitory system, leading to the reduced expression level of CGRP and subsequent bone loss [91].

The expression level of CGRP was elevated in the callus during bone fracture regeneration [92]. Genetic deletion of *α-CGRP* in mice impaired fracture healing by disturbing the distribution of callus bone cells [92, 93]. Multiple strategies to promote CGRP secretion have shown promising effects on fracture regeneration. After the implantation of magnesium-containing intramedullary nails, the expression of CGRP in the DRG increased significantly. More importantly, knocking down *CGRP* or its receptor decreased the periosteum’s capability for fracture healing [94]. Electrical stimulation of the DRG directly increased the biosynthesis and release of CGRP, therefore promoting bone fracture regeneration [95].

#### NGF-TrkA

NGF is a trophic factor that supports the development of specific sensory neurons both prenatally and postnatally. The effects of NGF are mediated through its interaction with TrkA receptor and the p75 neurotrophin receptor (p75NTR). A study using a mouse strain expressing a mutant TrkA protein (TrkA^F592A^) investigated the role of TrkA in bone development. Inhibition of TrkA impaired embryonic bone innervation, vessel formation, and subsequent bone formation [96, 97]. As the NGF signaling was mainly expressed in sensory nerves, inhibiting NGF-TrkA signaling has been shown to decrease mechanical loading-induced bone formation [98]. The level of NGF reduction was associated with increased levels of bone turnover markers [99]. Deletion of p75NTR led to decreased bone mass in mice, characterized by reduced osteogenic differentiation [100]. Given the involvement of NGF-TrkA in pain sensation, it is essential to explore the relationship between bone pain and bone formation [101, 102].

During the process of fracture healing, the activity of NGF was elevated and reached a peak at 3 d post-fracture [103]. NGF was secreted from different tissues or cell types, such as inflammatory cells, endothelial cells, and pericytes, following a fracture, thereby promoting the sprouting of TrkA-positive sensory nerves [103]. NGF deletion in myeloid cells resulted in reduced fracture healing ability, potentially by disturbing nerve ingrowth and cell migration [104, 105]. Inhibiting TrkA by a selective agonist, gambogic amide might contribute to the healing process of fracture [106]. A recent study suggested that NGF receptor played a critical role in regulating the bone remodeling process during osteoarthritis pathogenesis. NGF receptor deficiency in mice reduced the subchondral bone thickening and ectopic bone formation, highlighting its important role in bone formation [107].

#### Brain-derived neurotrophic factor (BDNF)-tropomyosin receptor kinase B (TrkB)

BDNF is a member of the neurotrophin family and shares some common features with NGF. The majority of BDNF was expressed in the CNS, while there was a proportion found in the peripheral nervous system, particularly in sensory nerous [108]. The primary receptor for BDNF is TrkB, although BDNF could also interact with the p75NTR, similar to other members of the neurotrophins family [109]. Genome-wide association studies have identified an association between the *BDNF* gene and vertebral BMD [110, 111]. Heterozygous mutation in the *BDNF* gene resulted in increased bone turnover and bone loss in mice [112]. Furthermore, 7,8-dihydroxyflavone, a TrkB agonist, activated TrkB and mitigated bone loss in either wild-type or BDNF^±^ mice by inhibiting osteoclast activity [113].

The level of *BDNF* mRNA elevated during the first week post-fracture [114]. BDNF promoted osteoblast migration and angiogenesis, therefore contributing to fracture healing [115, 116]. These effects were dependent on the phosphorylation and activation of TrkB [115]. However, another study suggested that inhibiting TrkB activity by a selective TrkB agonist impaired callus formation [117]. Therefore, while the role of BDNF-TrkB signaling in bone turnover was well-established, the precise mechanism underlying its involvement in fracture healing remained unclear and requires further exploration.

#### SP

SP is a member of the tachykinin family, encoded by the *Tac1* gene. The decreased level of SP has been observed in OP patients both locally and systematically [118]. Treatment with capsaicin to destroy sensory nerves decreased the SP content in these nerves, thus impairing bone formation and leading to reduced bone mass [119]. Blocking SP signaling prevented bone formation in either normal or osteoporotic mice [120]. Conversely, administration of SP improved bone microstructure [121, 122]. Global deletion of SP resulted in deteriorated bone microstructure, characterized by lower trabecular volume and thickness [123]. SP deletion significantly decreased bone formation while increasing bone absorption [123, 124]. Surprisingly, another study reported that SP deletion led to increased bone volume in the skull [125]. These seemingly contradictory observations suggested that the role of SP in bone homeostasis was complex and required further investigation.

The interaction between SP and NK1R played a crucial role in fracture healing. During the fracture healing process, SP-positive nerve fibers were observed in the callus and reached their peak on day 21 after the fracture. The density of SP-positive nerve fibers was positively correlated with the site of increased bone turnover rate [126]. This observation suggested that SP might have dual roles in both bone formation and resorption, effects that are influenced to some extent by mechanical loading. Blocking the NK1R with a selective antagonist abolished the interaction between NK1R and SP, thereby disrupting the fracture healing process [127]. Global deletion of the *Tac1* reduced bone formation during fracture healing, mainly because of decreased osteoblast number [123]. The mechanical strength of either fractured or intact limbs is impaired in Tac1^−/−^ mice. Further research found that Tac1^−/−^ mice exhibited an increased hypertrophic cartilage area 9 d post-fracture. These data indicated that the major regulatory role of SP in callus maturation was concentrated in the middle and late stages of fracture healing [128]. Another important function of SP in fracture healing was its mediation of post-fracture pain sensitization [70]. Incorporating SP into a chitin-polylactic-co-glycolic acid-calcium sulfate hydrogel promoted cell migration at bone defect sites and enhanced bone regeneration [129].

### Sympathetic nerve

#### NE

NE is the neurotransmitter released from the postganglionic fibers of the SNS. The receptor for NE can be divided into two categories, α-adrenergic receptors (α-AR) and β-adrenergic receptors (β-AR), each with distinct subtypes. Initially, it was believed that only β2-AR expressed on osteoblast mediated the efferent effect of leptin [130]. However, recent studies have shown that deletion of β-AR significantly improved bone mass, leading to the discovery that the nonselective β-blocker propranolol promoted bone formation and reduced fracture risk in humans and rodents [131, 132]. Global β2-AR knockout mice exhibited increased bone mass, suggesting that NE negatively regulated bone mass [133]. Nevertheless, other receptor subtypes also played a role in regulating bone mass through different mechanisms. For example, β1-AR knockout mice displayed lower femur BMD, bone volume/total volume, cortical size, and thickness. Moreover, double knockout of β1-AR and β2-AR reduced bone quality, distinct from the effects observed in β2-AR knockout mice [134]. Instead of β2-AR, β1-AR mediated the osteogenic differentiation of bone marrow mesenchymal stromal cells in mice with different sympathetic tone [135]. Both β1-AR and β2-AR were expressed in human bone tissue, but not β3-AR. Interestingly, selective activation of β1-AR increased BMD at the distal radius and decreased bone resorption markers such as C-terminal telopeptide [136]. Certain α-AR subtypes, including α1B-AR, α2A-AR, α2B-AR, and α2C-AR, were also expressed in osteogenic lineages [137, 138]. Double knockout of α2A-AR and α2C-AR led to sympathetic hyperactivity and high bone mass in the femur and vertebra [138]. In contrast, α1B-AR knockout mice exhibited decreased bone mass due to impaired bone formation [139]. Treatment with phenylephrine, an agonist for α1-AR, promoted the expression of CCAAT/enhancer-binding protein δ in osteoblasts. Based on these findings regarding the effects of different NE receptors on bone metabolism, selective AR-targeting drugs should be evaluated for potential clinical applications.

Large-scale clinical evidence suggested that the use of β-blocker was associated with a reduced risk of fractures [131]. The presence of α1-AR and β2-AR has been confirmed in the fracture callus tissue at 7, 14, and 21 d post-fracture [140]. Sympathetic nerve fiber ablation resulted in a delayed fracture healing process by increasing bone resorption and disturbing bony callus maturation [123, 128]. However, the changes in NE release during the fracture healing process remained largely unexplored. As a neurotransmitter, NE is released from the synaptic vesicles and binds to its receptor, inducing a post-synaptic response before being rapidly reabsorbed and cleared. Consequently, monitoring the temporal dynamics of NE during the fracture healing process poses significant challenges. Fortunately, advancements in neurotransmitter sensors might facilitate the in vivo assessment of NE activity [141].

#### NPY

NPY is predominantly synthesized by the SNS. NPY binds to its receptor, the neuropeptide Y receptor, which belongs to the family of G-protein coupled receptor. Among these, the NPY receptor 1 (Y1R) was highly expressed in the osteoblasts and other bone lineage cells, while Y2R, Y4R, Y5R, and Y6R exhibited limited expression in bone tissue [142]. However, Y2R, Y4R, Y5R, and Y6R, which are expressed in the hypothalamus, also play a regulatory role in bone homeostasis [142]. Research has shown that NPY knockout mice exhibited significantly increased bone mass, associated with transcriptional upregulation of runt-related transcription factor-2 and osterix in osteoblasts [27]. Conversely, treatment of osteoblasts with NPY resulted in decreased cAMP expression and impaired osteogenesis, which can be reversed by pharmacological blockade of Y1R [143]. Mice with Y1R ablation demonstrated an elevated bone mass phenotype, partially attributed to increased osteoblast activity [144–146]. Similarly, Y2R deletion in the hypothalamus resulted in a high bone mass phenotype via a central mechanism [147]. Notably, double knockout of Y1R and Y2R showed no additive effect on bone mass [145], while double knockout of Y2R and Y4R resulted in a greater increase in bone mass compared to single knockouts of Y2R or Y4R [148]. These observations suggested a complex influence of the NPY system on bone physiology, both centrally and peripherally.

The presence of NPY fiber in bone was observed in the fracture callus as early as 3 d post-fracture. The density of NPY-positive nerve fibers on the convex side of an angular fracture model negatively correlated with the callus thickness, indicating that the density of NPY nerve fibers was associated with callus remodeling [149]. The expression pattern of the NPY receptors also exhibited a time- and space-dependent manner. In a bone defect model, Y1R expression was upregulated in bone and DRG at an early stage (1 d post-injury), while Y2R in the hypothalamus was subsequently activated at a later stage (7 d post-injury) [150]. Administration of an NPY inhibitor attenuated the fracture healing process by downregulating extracellular signal-regulated kinase signaling and reducing macrophage aggregation [151]. Global and osteoblastic-specific Y1R deletion in mice resulted in delayed fracture healing, characterized by a decrease in bone callus volume and callus strength [152].

#### VIP

VIP is produced in the enteric neurons and other nerve terminals, particularly sympathetic nerve fibers [153]. VIP was widely expressed in the periosteum and bone, and sympathectomy leads to the loss of VIP-positive nerve fibers in the periosteum [19]. VIP binds to vasoactive intestinal peptide receptor (VPAC)1 and VPAC2, which belong to the G-protein coupled receptor family. VIP has been shown to mediate several important functions in the skeletal system, including bone maintenance and embryonic bone development [154, 155]. The serum VIP level was positively associated with BMD in postmenopausal women [156]. However, the role of VIP in bone metabolism remained controversial [157]. A previous study found that VIP bound to both osteoclasts and osteoblasts and was linked to the transient suppression of osteoclast activity [158], while another study demonstrated that VIP promoted bone resorption via the cAMP signaling pathway [159]. In a model of sympathectomized mice, VIP treatment could rescue the impaired fracture healing by increasing osteogenesis [160]. A similar osteogenic role of VIP was also demonstrated in a skull defect model, primarily through activation of the Wnt/β-catenin signaling pathway [161].

### Parasympathetic nerve

ACh serves as a neurotransmitter utilized by motor neurons, interneurons of the SNS, and postganglionic fibers of the parasympathetic nerves. There are two major types of ACh receptors, nicotinic and muscarinic. Deficits in ACh synthesis or receptor function might lead to altered bone mass in mice. For instance, heterozygous deficiency in the choline transporter has been shown to decrease vertebrae bone mass in young female mice [162]. Acetylcholinesterase (AChE) and butyrylcholinesterase are two enzymes responsible for hydrolyzing and degrading ACh following synaptic transmission. Knocking out AChE in mice decreased osteoclast numbers in vertebrae but also reduced trabecular parameters in L3 vertebrae and cortical area fraction in femur [163]. Conversely, deletion of butyrylcholinesterase led to increased trabecular and cortical quality as well as higher osteoclast numbers compared to wild-type mice [164]. Defects in ACh receptor function were also associated with altered bone metabolism. It was observed that nicotinic receptor α2nAchR-deficient mice exhibited decreased bone mass in the femur and vertebral body [83]. Kliemann et al. [165] compared the effects of muscarinic receptor M3R and nicotinic receptor α7 on bone quality. Their findings suggested that knocking out M3R, but not α7, decreased bone quality by reducing collagen I and II expression. Another study demonstrated that M3R deficiency increased osteoclast activity, as visualized by TRACP staining [166]. However, further studies indicated that loss of the α7 receptor might also lead to improved trabecular and cortical bone quality in mice [167, 168]. Furthermore, the nicotinic receptor α9 positively regulated bone mass by enhancing osteoblast activity without affecting osteoclasts [169]. Donepezil, a central-acting acetylcholinesterase inhibitor (AChEI), has been shown to upregulate parasympathetic nerve tone and increase bone mass [170]. Collectively, these observations suggested that ACh generally acted as a positive regulator of bone quality. However, the specific functions of different ACh receptor subtypes required further clarification.

The role of ACh and AChEIs in fracture healing remained controversial. In a rat model of tibial defect, administration of AChEIs was found to delay bone healing and implant osseointegration processes [171]. However, AChEIs have been shown to reduce the incidence of fractures in patients with cognitive impairment [172, 173]. Furthermore, AChEIs might enhance fracture healing by promoting bone union in Alzheimer’s disease patients compared to non-users [174]. The exact mechanisms and the dual role of ACh and AChEIs in both central and peripheral systems remain to be elucidated.

### The crosstalk between sensory nerve and sympathetic nerve

Although evidence has shown that different cell markers and molecules were crucial in specific nerve fibers or terminals, the regulatory effects of individual nerve types were not isolated [175–177]. In adipose tissue, ablation of sensory nerve terminals enhanced lipogenesis and thermogenesis when the sympathetic nerve remained intact [178], suggesting that the sensory nerves acted as a brake on the sympathetic system. Another study demonstrated that sympathetic and sensory nerve tone synergized to regulate osteoblast activity and mesenchymal stem cell differentiation through the prostaglandin E2 signaling pathway [179]. Generally, sympathetic nerves antagonize parasympathetic nerves. Moreover, certain markers originally thought to be specific to particular nerve terminals exhibited overlap with other neuronal subtypes [103, 180, 181].

### Other CNS signalings that affect bone homeostasis

#### Leptin

Leptin is a hormone primarily produced by adipocytes and plays a crucial role in regulating energy balance. The regulatory effect of leptin on bone mass has been identified in leptin and its receptor deficiency in ob/ob and db/db mice [182]. Although both db/db and ob/ob mice exhibited a low bone mass phenotype, leptin did not directly affect osteoblast activity [183]. Infusion of leptin into the third ventricle resulted in a low bone mass phenotype [184]. These findings suggested that leptin mediated its effects on bone via a CNS-dependent mechanism. Subsequent research demonstrated that leptin’s central regulation of bone mass did not rely on anorexigenic neuropeptides such as agouti yellow and melanocortin 4 receptor. Instead, genetic or pharmacological inhibition of β-adrenergic signals led to a high bone mass resistant to leptin treatment [130]. Moreover, leptin regulated bone remodeling through two distinct pathways: the SNS, which controlled osteoclast activity, and cocaine- and amphetamine-regulated transcript (CART), which influenced osteoblast activity [133]. Notably, leptin also exerted its regulatory effects on bone metabolism via peripheral mechanisms. Increased leptin levels might directly stimulate cortical bone formation through a pro-osteoblast effect, while decreased trabecular bone mass via a CNS-dependent mechanism [185]. Subcutaneous and intracerebroventricular leptin injection increased BMD, mineral apposition rate and bone area [183]. However, genetic overexpression of leptin in the hypothalamus led to lower trabecular bone volume 5 weeks after vector administration but was restored after 10 weeks [186]. These observations suggested that leptin had distinct roles in both peripheral and central regulation of bone metabolism.

Leptin signaling has been shown to mediate the function of stem cells during the fracture healing process. Loss of LepR in limb bone marrow stromal cells accelerated the bone fracture healing process [187]. Leptin bound to its receptor LepR and activated the Janus-activated kinase 2/singal transducers and activators of transcription 3 signaling pathway, thus enhancing osteogenesis. More recently, it has been found that LepR-positive bone marrow skeletal stem cells specifically repaired drill injuries but did not contribute to bicortical fracture healing [188]. Leptin signaling also mediated the facilitation effect of traumatic brain injury (TBI) on fracture healing. In rats with both fracture and TBI, the elevated leptin levels were comparable to those observed in animals with either condition alone [189]. Moreover, the accelerated fracture healing induced by TBI was abolished in leptin-deficiency mice due to reduced osteocalcin levels [190]. In addition to TBI, spinal cord injury could also elevate leptin levels, subsequently promoting callus formation [191]. Besides the direct effects of circulating leptin on bone fracture healing, the role of leptin-regulated fracture healing via the hypothalamus-SNS circuit remained to be elucidated.

#### Neuromedin U (NmU)

NmU is a highly conserved neuropeptide that is involved in the regulation of smooth muscle contraction, blood pressure, feeding and energy homeostasis, and ion transport [192]. NmU is widely expressed across various tissues, with the highest expression levels observed in the gastrointestinal tract and CNS. The effects of NmU are mediated by two G-protein coupled receptors: NmUR1, which primarily acts on peripheral organs, and NmUR2, which is predominantly found in the CNS. In the CNS, NmU and NmUR2 were concentrated in the hypothalamus. Polymorphisms in NmU have been associated with bone quality in children [193]. Of note, NmU-deficient mice exhibited a high bone mass phenotype owing to increased bone formation [194]. Importantly, NmU was not detected in the bone tissue and had no direct effect on osteoblasts. Instead, its influence on bone mass was mediated via the CNS. Furthermore, the pro-osteogenesis effects of NmU did not depend on leptin or peripheral sympathetic tone [194]. Additional studies investigating site-specific knockdown of NmU in the hypothalamus and NMUR1 knockout did not identify any observable bone phenotypes [195]. Collectively, these observations suggested that the role of NmU in bone metabolism was multifaceted.

#### Endocannabinoids

Endogenous cannabinoids, or endocannabinoids, were first discovered due to the high affinity of tetrahydrocannabinol (THC) for brain receptors. The endocannabinoid system includes two major cannabinoid receptors, cannabinoid receptor type 1 (CB1, encoded by *Cnr1* gene), primarily expressed in the CNS, and cannabinoid receptor type 2 (CB2, encoded by *Cnr2* gene), predominantly found in the peripheral organs [196]. The endocannabinoid system participates in multiple biological processes, including learning, memory, eating, and anxiety regulation [197, 198]. Global CB2 deficiency in mice led to a low trabecular and cortical bone mass phenotype due to impaired osteoblast differentiation [199]. The role of the CB1 in bone varied depending on the mouse strain [200]. In CD1 background mice lacking CB1 (CD1^CB1−/−^), male mice exhibited high bone mass, while female mice showed no significant difference in bone quality. In contrast, in C57BL/6 J background mice lacking CB1 (C57^CB1−/−^), both male and female mice exhibited low bone mass and increased osteoclast activity. However, another study found that CB1 loss resulted in decreased cortical bone mass in both male and female mice, while increased trabecular bone mass was observed only in female mice [201]. In CB1/2 double knockout mice, bone accrual was accelerated in neonates, and adult mice exhibited improved trabecular bone mass. Mechanically, CB1/2 deficiency reduced osteoclast number and expression of osteoclast-related genes [202]. Collectively, these observations indicated that CB1/2 might have distinct functions in regulating bone homeostasis, with effects varying by site, gender, or genetic background. Further exploration of these mechanisms is warranted.

The osteogenesis process was promoted by cannabinoid treatment, suggesting its role in regulating fracture healing. Endocannabinoid tone was significantly activated during osteoblast differentiation [203]. Moreover, activation of the CB2 promoted osteogenic differentiation via a p62-dependent mechanism [204]. However, there were limited in vivo studies investigating the role of endocannabinoids during the fracture healing process. A study by Kogan et al. [205] found that non-psychotropic cannabis cannabidiol (CBD), but not THC, improved biomechanical properties during bone fracture healing. Notably, neither THC nor CBD affected bone mineral content or callus size. The underlying mechanism might be attributed to cannabis compounds regulating collagen crosslinking during the late phases of healing.

#### Serotonin

Serotonin (5-hydroxytryptamine, 5-HT) is a monoamine neurotransmitter distributed in the CNS and enteric nervous system (ENS). In the CNS, serotonin participated in the regulation of mood, addictive behavior, and feeding. In the ENS, the serotonin network is essential for motility, blood flow, and intestinal flora regulation [206]. The majority of circulating serotonin was produced by the enterochromaffin (EC) cells in the ENS [207]. Serotonin played a crucial role in bone homeostasis, with distinct functions at central and peripheral levels [208]. Tryptophan hydroxylase 1, the rate-limiting enzyme in serotonin synthesis, negatively regulated bone mass by stimulating osteoclast function [209, 210]. Low-density lipoprotein receptor-related protein 5 (Lrp5) is another key regulator of serotonin synthesis in the EC cells. Loss-of-function mutations in Lrp5 led to low bone mass, while gain-of-function mutations resulted in high bone mass [211]. Lrp5 could affect bone homeostasis directly [212], or indirectly through an EC cell-dependent mechanism [32]. Mechanically, Lrp5 inhibited tryptophan hydroxylase 1 expression, thereby reducing serum serotonin levels [32]. A recent work discovered that Piezo1 in intestinal epithelial cells downregulated bone mass via a serotonin-dependent pathway [213]. In addition to its role in serotonin production, bone mass was also influenced by different 5-HT receptor subtypes. Ablation or inhibition of 5-HT2A and 5-HT2B resulted in decreased bone mass due to reduced osteoblast differentiation [210, 214]. Conversely, deletion of the 5-HT1B increased bone mass by upregulating osteoblast number [215].

Additionally, as a neurotransmitter, serotonin in the CNS exerts distinct effects on bone mass [208]. Tryptophan hydroxylase 2 (Tph2) is a neuron-specific tryptophan hydroxylase, with the highest expression in the raphe nucleus. Studies on *Tph2* knockout mice have revealed different and somehow contradictory bone phenotypes: decreased trabecular bone mass in vertebrae and femur [32], as well as elevated vertebrae trabecular bone with reduced femoral cortical thickness [216]. A study by Yadav et al. [32] identified serotonin neuron projections from the dorsal raphe, median raphe, and VMH. In the hypothalamus, multiple serotonin receptor subtypes were widely expressed, with the Htr2c showing the highest expression levels. Knockout of *Htr2c* decreased bone mass, while re-expression of Htr2c under the SF1 promoter in VMH partially restored bone mass loss. Moreover, serotonin regulates bone mass via a CREB-dependent mechanism in the hypothalamus [31].

The inhibitory effects of serotonin and selective serotonin reuptake inhibitors (SSRI) on fracture healing have been observed in several clinical studies [217–219]. SSRI and serotonin impaired bone fracture healing by decreasing osteogenic differentiation and mineralization [220]. The adverse effects of SSRI on fracture healing could be reversed by β-blocker administration, suggesting the involvement of the SNS [221]. However, the functional discrepancies of serotonin between the CNS and serum in regulating the bone fracture healing process, as well as the specific serotonin receptor subtypes involved, remained unclear.

#### CART

CART is a neuropeptide encoded by the *CARTPT* gene. Initially discovered in the striatum, CART exhibits the highest expression levels in the hypothalamus. CART acted on various brain nuclei to mediate distinct biological functions: ARC and VMH for feeding regulation, the dorsomedial nucleus and lateral hypothalamus for body weight, and the ventral tegmental area for reward processing [222]. Global knockout of CART decreased bone mass, mainly through upregulating osteoclast activity [133]. Moreover, ex vivo studies showed that CART-deficient bone marrow macrophages exhibited no impairment in osteoclast differentiation, suggesting a central role of CART in bone homeostasis. In contrast, elevated serum CART level has been identified as a factor contributing to high bone mass [223, 224]. Exogenous CART administration elevated bone mass, while OVX abolished this effect [225]. In the CNS, genetically targeted AP1 activation in CART-positive neurons promotes trabecular bone formation [29]. However, overexpressing of CART in hypothalamic neurons did not result in increased bone mass [224]. These observations suggested that CART neurons in the brain controlled bone mass via a more complex pathway rather than acting as a neurotransmitter or releasing CART into the bloodstream.

#### Circadian rhythm

The circadian rhythm refers to the biological rhythm that meets the following criteria: an endogenous cycle of approximately 24 h, entrainable by external cues, and exhibiting temperature compensation [226]. The circadian rhythm is controlled by the core molecular circadian oscillator, consisting of transcription factors BMAL1/CLOCK, along with their negative repressor proteins cryptochrome circadian regulator 1 (CRY1) and Period (PER). The BMAL1-CLOCK transcriptional complex promoted the transcription of CRY and PER, which in turn inhibited the transcriptional activity mediated by BMAL1-CLOCK. In addition to the central circadian clock, supplementary regulators such as retinoic acid-related orphan receptor (ROR) and nuclear receptor subfamily 1 group D (NR1D1/2 or REV-Erb) also exhibited circadian oscillation and participated in circadian control [227].

In mammals, the suprachiasmatic nuclei (SCN) served as the central pacemaker of the body, while nearly all cells possess their circadian clock [228]. Disruption of the circadian rhythm exerted a significant impact on bone mass via different mechanisms [229, 230]. Simply shifting the light–dark circle alters bone microstructure in the long term, suggesting that maintaining a normal circadian rhythm is crucial for bone health [231]. In the CNS, Y6R loss in the SCN resulted in reduced cortical and trabecular bone mass in axial and appendicular bones by inducing an osteoblast-osteoclast imbalance [232]. Deletion of sine oculis 3 in the Synapsin-Cre allele disturbed circadian output from the SCN, subsequently decreasing bone mineral content and density [233]. Melatonin, a hormone secreted from the pineal gland, exhibited circadian secretion patterns with peak concentrations at night and lower concentrations during the day [234]. The circadian rhythm of the skeletal system could be modulated by fluctuation in melatonin levels [235].

Interfering with peripheral circadian signaling in the skeletal system exerts distinct effects on bone mass. Global deletion of BMAL1 led to reduced long bone length and decreased trabecular and cortical bone quality [236]. BMAL1 deficiency resulted in decreased bone mass in mandibles by downregulating osteoprotegerin levels [237]. Osteoclast-specific BMAL1 deletion using Ctsk-Cre in mice exhibited a high bone mass phenotype [230], although other studies suggested Bmal1 might not directly affect osteoclast activity [236]. Disturbance of circadian rhythm by knocking out BMAL1 in the intestine [238] and colon [239] resulted in bone loss, possibly through increased sympathetic tone [238]. Knocking out *Clock* gene led to an elongated circadian rhythm in mice [240]. Mice carrying *Clock* mutant allele (Clock^Δ19^ mice) exhibited low bone mass due to reduced bone formation [241]. The mechanism underlying CLOCK/BMAL1’s control of bone homeostasis might be attributed to the transcriptional regulation of downstream genes.

PER and CRY proteins, acting as the negative regulator of BMAL1/CLOCK complex, also participated in the regulation of bone mass. Single knockouts of PER1 or PER2 had no observable bone phenotype, but PER1/2 double knockout resulted in a high bone phenotype. Moreover, deficiency in PER protein led to dysregulated circadian gene expression, which was tightly controlled by sympathetic signaling [234]. Another study suggested that mice carrying a *Per2* mutant allele exhibited increased bone mass [242]. Research has also highlighted the role of CRY in bone metabolism. Both *Cry2* knockout and *Cry1/2* double knockout mice showed a high bone mass phenotype [234, 243], although no specific reports existed on the effect of *Cry1* deficiency on bone phenotype.

ROR and REV-Erb are both members of the nuclear receptor family. They control the transcription activity of BMAL1 via retinoic acid receptor-related orphan receptor response element [227]. Rora mutant mice, known as Staggerer mice (sg mice), have shortened locomotor activity rhythms. These mice exhibited disturbed bone homeostasis, characterized by osteopenic long bone and decreased osteogenesis [244]. Interestingly, knocking out Rorb, another member of the ROR family, resulted in an elongated circadian rhythm and increased bone mass [245, 246]. A previous study found that a REV-Erb agonist reversed bone loss in OVX-induced osteoporotic mice by suppressing osteoclastgenesis [247]. However, to date, there is no genetically modified animal model that confirmed REV-Erb’s direct participation in bone metabolism.

## New approach integrating nerve function and bone homeostasis

### Targeting nerves to alleviate OP

OP is characterized by low bone mass and microarchitectural deterioration due to various etiologies. Both peripheral nerve and central nerve signals can regulate bone homeostasis via distinct mechanisms. Among these bone-regulating nerve signals, the translational potential of sympathetic tone and its regulator β-blocker has attracted significant attention [20]. The effects of different β-blocker (propranolol, atenolol, and nebivolol) on BMD and bone turnover markers have been evaluated in patients, indicating the protective role of β-blockers in bone metabolism [136]. Given that β-blockers showed relatively weak anti-osteoporotic effects with fewer side effects compared to traditional anti-osteoporotic medications (e.g., bisphosphonates, denosumab, and teriparatide), they might be suitable for early prevention of OP. In addition to β-blockers, other nervous system-modulating medications, neuropeptides, neurotransmitters, and optoelectronic methods also exhibited potential for preventing and treating OP [248, 249]. However, more mechanistic and clinical evidence is needed to support these approaches.

### Promoting osseointegration

Osseointegration refers to the integration process of forming direct structural and functional connections between the implant surface and bone tissue [250]. Successful osseointegration required the participation of intact nerve function. Neurectomy or botox-induced muscle paralysis impaired titanium implant osseointegration [251], suggesting that both sensory and sympathetic nerves played crucial roles in this process [252]. Titanium implants promoted osseointegration via α2-adrenergic receptors, and the peripheral circadian molecule Npas2 positively regulated this process [253]. Another study found that microelectrode stimulation of sympathetic nerves significantly enhanced the implant-bone osseointegration process [254]. More recent advancements have integrated mechanical and neural interfacing in bionic prosthetic limbs, combining muscle function, osseointegration, and implanted sensors, which sheds light on future applications for nerve-assisted functional recovery [255, 256].

### Accelerating fracture healing

The fracture healing process requires the intimate coordination of different nerve fiber types. As mentioned in the previous part, nerve function is critical for promoting fracture healing and preventing recurrent fractures. Therefore, an increasing number of studies have explored the use of nerve factors or nerve stimulation to enhance bone fracture repair and improve local nerve function [95, 257, 258]. Our previous work demonstrated that magnesium implants promoted fracture healing by enhancing CGRP release [94]. In a subsequent study, an electric stimulator inserted into the DRG neuron accelerated osteoporotic fracture healing by promoting CGRP release [95]. A well-designed photosensitive conductive hydrogel containing magnesium-modified black phosphorus facilitates CGRP nerve fiber regeneration and neurite outgrowth, thereby achieving optimal innerved bone regeneration [259]. Microparticles containing β-nerve growth factor (β-NGF) or local injection of β-NGF promoted endochondral ossification during fracture healing [260, 261]. In a chronic psychosocial stress model, administration of sympathetic nerve inhibitor β-blocker improved callus formation [262]. Neuromodulation therapies, including peripheral nerve stimulation, transcranial magnetic stimulation, transcranial direct current stimulation, and transcutaneous electrical nerve stimulation, are viable for alleviating pain at different sites [38]. It is worth exploring the application of these neuromodulation techniques in fracture healing and related pain management.

## Conclusions and future perspectives

In this review, we summarized evidence and animal experimental studies that linked both central and peripheral nervous systems with bone homeostasis. Moreover, many studies unveiled the underlying molecular, cellular, and circuit mechanisms of these phenomena. The nervous system tightly regulates bone homeostasis in both physiological and pathological conditions. From a translational perspective, recent works have focused on integrating bone regeneration with nerve function, particularly motor function and pain control. Novel biomaterials facilitated the regeneration or repair of bone defects and fractures by promoting nerve ingrowth and bone re-innervation. In addition to local factors and implants, innovative techniques such as transcranial magnetic stimulation, epidural electrical stimulation, and electrode recording, and optogenetic modulation provide non-pharmacological and minimally invasive methods to modulate nerve function.

However, many nerve-governed pathways to the skeletal system remain poorly understood, and a comprehensive understanding of how the nervous system transmits brain signals to its peripheral effector, bone, is still lacking. The immediate or short-term effects of the nervous system were often easily observable and measurable (e.g., heart rate, gut movement, and body temperature). In contrast, bone remodeling is a relatively long-term physiological process, which presents challenges in fully understanding the immediate and dynamic nerve effects on maintaining relatively static bone homeostasis. Of note, the regulatory role of the nerve-bone interface is not unidirectional, accumulating evidence suggests that bone-derived factors can also influence nerve function. Nonetheless, the development of new techniques holds promise for uncovering a more precise role of nerves in the skeletal system.

## Data Availability

Not applicable.

## Abbreviations

5-HT: 5-Hydroxytryptamine;
α-AR: α-Adrenergic receptors
β-AR: β-Adrenergic receptors
ACh: Acetylcholine
AchE: Acetylcholinesterase
AchEI: Acetylcholinesterase inhibitor
AgRP: Agouti-related peptide
AP1: Activator protein 1
ARC: Arcuate nucleus
AVP: Arginine vasopressin
BDNF: Brain-derived neurotrophic factor
BLA: Basolateral complex of the amygdala
BMD: Bone mineral density
cAMP: Cyclic adenosine monophosphate
CART: Cocaine- and amphetamine-regulated transcript
CB: Cannabinoid receptor
CBD: Cannabidiol
CeA: Central nucleus of the amygdala
CGRP: Calcitonin gene-related peptide
CNS: Central nervous system
CREB: Cyclic adenosine monophosphate response element-binding protein
DRG: Dorsal root ganglion
EC: Enterochromaffin cell
ENS: Enteric nervous system
FoxO1: Forkhead box O1
Htr2c: 5-Hydroxytryptamine receptor 2C
LC: Locus coeruleus
LepR: Leptin receptor
Lrp5: Low-density lipoprotein receptor-related protein 5
NE: Norepinephrine
NGF: Nerve growth factor
NK1R: Neurokinin 1 receptor
NmU: Neuromedin U
NPY: Neuropeptide Y
OP: Osteoporosis
OVX: Ovariectomy
p75NTR: P75 neurotrophin receptor
PDK1: Pyruvate dehydrogenase kinase 1
POMC: Proopiomelanocortin
PVN: Paraventricular hypothalamic nucleus
SCN: Suprachiasmatic nuclei
SF1: Steroidogenic factor 1
SNS: Sympathetic nervous system
SP: Substance P
SSRI: Selective serotonin reuptake inhibitors
TBI: Traumatic brain injury
TH: Tyrosine hydroxylase
THC: Tetrahydrocannabinol
TrkA: Tropomyosin receptor kinase A
TrkB: Tropomyosin receptor kinase B
VAChT: Vesicular ACh transporter
VIP: Vasoactive intestinal peptide
VMH: Ventromedial hypothalamus
Y1R: NPY receptor 1
Y2R: NPY receptor 2
